# Operationalizing the CARE and FAIR Principles for Indigenous data futures

**DOI:** 10.1038/s41597-021-00892-0

**Published:** 2021-04-16

**Authors:** Stephanie Russo Carroll, Edit Herczog, Maui Hudson, Keith Russell, Shelley Stall

**Affiliations:** 1 Native Nations Institute at the Udall Center for Studies in Public Policy, University of Arizona, Tucson, Arizona USA; 2 College of Public Health, University of Arizona, Tucson, Arizona USA; 3 Vision & Values, Brussels, Belgium; 4 Te Kotahi Research Institute, University of Waikato, Hamilton, New Zealand; 5 Australian Research Data Commons, Caulfield East, Victoria, Australia; 6 Data Leadership, American Geophysical Union, Washington, D.C. USA

**Keywords:** Ethics, Policy, Institutions

## Abstract

As big data, open data, and open science advance to increase access to complex and large datasets for innovation, discovery, and decision-making, Indigenous Peoples’ rights to control and access their data within these data environments remain limited. Operationalizing the FAIR Principles for scientific data with the CARE Principles for Indigenous Data Governance enhances machine actionability and brings people and purpose to the fore to resolve Indigenous Peoples’ rights to and interests in their data across the data lifecycle.

## Introduction

This discussion emerged from two joint, virtual conference sessions that integrated parallel processes at the Research Data Alliance (RDA): “Operationalising Be FAIR and CARE” (https://www.rd-alliance.org/operationalising-be-fair-and-care) and “Implementing the CARE Principles: The CARE-full Process” (https://www.rd-alliance.org/implementing-care-principles-care-full-process). The FAIR Data Maturity Model Working Group, hosted by the Research Data Alliance, was endorsed for activity in September 2018 with the primary objective to develop a core set of criteria that would be used across all the existing implementation methods for FAIR Data Principles^1^ (see https://www.rd-alliance.org/group/fair-data-maturity-model-wg/case-statement/fair-data-maturity-model-wg-case-statement). With this common set of criteria, outcomes of different assessment methods could be compared and provide a better understanding of the true status across the research community as we move towards meeting the criteria defined. Following a year-long public discussion and building on Indigenous Data Sovereignty network activities, the Global Indigenous Data Alliance released the CARE Principles for Indigenous Data Governance in September 2019^2^. After the launch of the CARE Principles, the RDA International Indigenous Data Sovereignty Interest Group, which produced the CARE Principles, and the RDA FAIR Data Maturity Model Working Group joined together to begin to explore how to operationalize FAIR with CARE. This manuscript briefly introduces the CARE Principles and describes CARE in the context of scientific data, FAIR in the context of Indigenous data, how FAIR and CARE intersect, and implications and next steps for actionable change toward operationalizing FAIR with CARE.

## The CARE Principles

Indigenous data are data, information, and knowledge, in any format, that impact Indigenous Peoples, nations, and communities at the collective and individual levels; data about their resources and environments, data about them as Individuals, and data about them as collectives^3,4^. Indigenous Data Sovereignty draws on the United Nations Declaration on the Rights of Indigenous Peoples (UNDRIP), which reaffirms the rights of Indigenous Peoples to control data about their peoples, lands, and resources^5,6^. Indigenous data governance enacts those rights through mechanisms grounded in Indigenous rights and interests that promote Indigenous values and equity, while providing a framework for addressing deeper historical issues associated with barriers for underrepresented communities and knowledge systems^7^.

The ‘CARE Principles for Indigenous Data Governance’ address concerns related to the people and purpose of data; Collective benefit, Authority to control, Responsibility, and Ethics, and their respective sub-principles^2^. The CARE Principles detail that the use of Indigenous data should result in tangible benefits for Indigenous collectives through inclusive development and innovation, improved governance and citizen engagement, and result in equitable outcomes^3^. **Collective benefit** is more likely to be realized when data ecosystems are designed to support Indigenous nations and when the use/reuse of data for resource allocation is consistent with community values. UNDRIP asserts Indigenous Peoples’ rights and interests in data and their **authority to control** their data. Access to ‘data for governance’ is vital to support self-determination and Indigenous nations should be actively involved in ‘governance of data’ to ensure ethical reuse of data. Given the majority of Indigenous data is controlled by non-Indigenous institutions there is a **responsibility** to engage respectfully with those communities to ensure the use of Indigenous data supports capacity development, increasing community data capabilities, and the strengthening of Indigenous languages and cultures. Similarly, Indigenous Peoples’ **ethics** should inform the use of data across time in order to minimize harm, maximize benefits, promote justice, and allow for future use.

The CARE Principles are designed to be complementary to the FAIR Principles, Findable, Accessible, Interoperable, Reusable^1^, and other mainstream data frameworks, and promote equitable participation and outcomes from data access, use, reuse, and attribution in contemporary data landscapes^2^. Given the tension between protecting Indigenous rights and interests in data while encouraging FAIR data in a global research environment that also supports open data^8^, implementation of the CARE Principles should be seen as a required dimension of open and FAIR data that ensures the use of data aligns with Indigenous rights, is as open as determined by Indigenous communities, is purposeful, and enhances the wellbeing of Indigenous Peoples.

## The FAIR Data Maturity Model

In the two years following the publication of the FAIR Data Principles in 2016^1^, numerous efforts across the scientific ecosystem developed assessment methods for their disciplines using different interpretations and measurement criteria. This diversity is both a benefit to the communities trying to implement the FAIR criteria, but also a challenge due to the inability to easily compare outcomes. In 2018, the FAIR Data Maturity Model working group identified thirteen FAIR data assessment methods being used or in development. Through an iterative process of 18 months, representatives of these assessment methods and others worked to develop an initial framework that each assessment method could use to support comparison of outcomes. The authors believe similar methods could be used to develop a framework for the CARE Principles in an equivalent 18-month timeframe using the RDA community and platform as a host for the effort. To give more context, FAIR was initially conceived in 2012 with the endorsed FAIR Data Maturity Model Framework recommendations completed in 2020, a duration of 8 years. The development of a maturity model framework for CARE could be accelerated with completion in as little as 4 years from the inception of the CARE Principles, targeting the year 2022 if all efficiencies were in place.

## Operationalizing CARE and FAIR

**Fig. 1:**
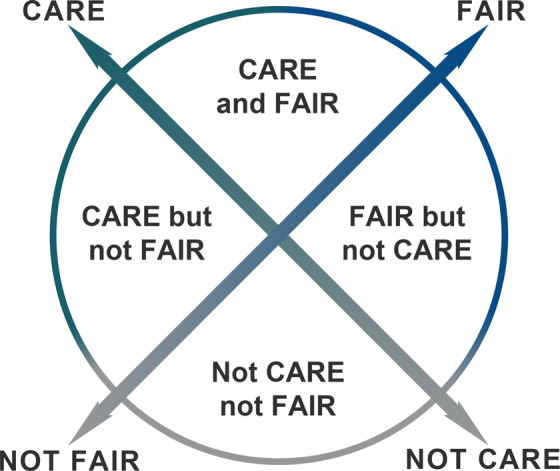
Operationalizing Complementary Principles: the Range of Be FAIR and CARE.

Today there is a paradox of scarcity and abundance for Indigenous data^7,9^. There is a scarcity of data that align with Indigenous rights and interests and which Indigenous Peoples can control and access in a manner consistent with the CARE Principles. There is an abundance of data that are buried in larger collections, hard to find, mislabelled, and controlled (legally and literally) by others in a manner inconsistent with the FAIR and CARE Principles^10^. These two trajectories are represented in Fig. 1 which illustrates that data could be subject to both CARE and FAIR and create different outcomes depending on how implementation criteria have been operationalized within cyber infrastructures.

It is apparent the development process for CARE should necessarily align with FAIR given the various ways in which the principles might intersect around different datasets or contexts. Figure 1 outlines the range of possibilities where FAIR and CARE might currently be applied to data resources. A range of datasets are working towards being FAIR however many have not taken active steps to towards adopting mechanisms that support CARE. The Earth Science Information Partners (ESIP) are working toward developing guidance on how to operationalize CARE and FAIR for repositories. Earth and space science research data is highly relevant to Indigenous communities. ESIP’s Sustainable Data Management Cluster is currently reviewing the role of repositories for both CARE and FAIR to determine the needed operational services and other criteria. An early observation from this work is that data that are well documented through compliance with FAIR criteria are easier to manage when implementing the CARE Principles.

Tribal databases and Indigenous Content Management Systems (e.g. Mukurtu.org) hold tribal data using protocols consistent with tribal values and worldviews, thus employing CARE. However, these collections are generally not consistent with FAIR principles and require enriched metadata and protocols. The Integrated Data Infrastructure (IDI) in New Zealand has developed a data access protocol called Ngā Tikanga Paihere which is based on Indigenous concepts and values consistent with CARE as well as ensuring the data are also FAIR (https://data.govt.nz/use-data/data-ethics/nga-tikanga-paihere/). Application of CARE with FAIR requires a clear set of criteria and tools such as the FAIR Data Maturity Model. Compiling existing and creating new tools and criteria for implementing the CARE Principles are needed to achieve data that are FAIR with CARE.

## CARE in the Context of Scientific Data

The FAIR Principles are aligned to the global shift towards open science and open data, promoting data centric criteria that facilitate increased data sharing among entities while ignoring relationships, power differentials, and the historical conditions associated with the collection of data^10,11^. These factors continue to affect how ethical and socially responsible data use can occur within the sciences particularly as machine learning approaches accelerate data re-use.

Concerns about secondary use of data, problems with bias and social inequity, and limited opportunities for benefit-sharing, have focused attention on the tension that Indigenous communities feel between protecting their interests in scientific data generated from their lands, waters, and people, while supporting, or being subject to open data and data sharing initiatives^8,12^. Indigenous communities face a number of challenges in facilitating the culturally appropriate reuse of scientific data.

The CARE Principles should not be thought of as only applying to Indigenous Knowledge or Traditional Knowledge but also scientific data. The CARE Principles speak to how scientific data are used in ways that are purposeful and oriented towards enhancing wellbeing of people. The CARE Principles are likely to find expression across the data lifecycle from collection to curation, from access to application, with implications and responsibilities for a broad range of entities from funders to data users. Our expectation is that through the development of an implementation process for CARE we will develop criteria for cultural metadata, provenance, Indigenous governance, Indigenous ethics, transparency, integrity, and equity.

It is important to note that in many cases scientific datasets also contain Indigenous Knowledge. Users and producers of such data, both in the past and currently, often do not realize the need to acknowledge Indigenous provenance or make space for cultural metadata when creating this data. One tool that predates yet exemplifies the combined practice of CARE and FAIR data are the Traditional Knowledge (TK) Labels, an extra-legal digital mechanism that re-positions Indigenous cultural authority and governance over Indigenous data and collections. The TK Labels are digital tags which restore relationships between Indigenous and non-Indigenous rights holders by correcting and providing more information about cultural material collected, often in circumstances of duress and with questionable consent^13,14^.

The TK Labels improve the quality of provenance, encourage communities to enrich records with their own traditional knowledge, and increase capacity for better understanding of equity and decision-making regarding re-use and circulation. As the TK Labels are directly incorporated into the digital infrastructure of catalogue, classification, and content management systems they work at the level of metadata to enhance and legitimize locally based decision-making and Indigenous governance frameworks for determining ownership, access, and culturally appropriate conditions for sharing historical and contemporary collections of cultural heritage. TK Labels are in use in a number of institutions including the Library of Congress^15^. By enabling local authority and reflecting community protocols the TK Labels are a good example of a tool that supports implementation of both FAIR and CARE Principles.

The CARE Principles and tools like the TK Labels have important implications for international policy frameworks like the Convention on Biological Diversity and the Nagoya Protocol. The Nagoya Protocols supports fair and equitable benefit sharing for genetic resources but currently excludes digital sequence information, in part due to the practicalities of tracking provenance and secondary use of datasets^16^. While open genomic data supports innovation it also enables the appropriation of value and increases inequities^12^. The implementation of CARE, and application of TK Labels, to genomic databases or metadata databases like GEOME will illustrate the value that this work can contribute towards delivering on the promise of fair and equitable benefit sharing^17^.

Additionally, there has been broadening interest in applying the CARE Principles outside the realm of Indigenous Peoples. The idea that specific communities could contribute to the development of protocols that inform the ethical use of data about them resonates with the CARE Principles, addressing concerns about fairness, trust, and accountability that are increasingly being advanced and by allowing contributors, as collectives, to have a say in how their data actually gets used^10^. However, in the development phase, the implementation of the CARE Principles will focus on the ethical and appropriate collection and use of Indigenous data, to allow for the full contribution of Indigenous values and perspectives in the innovation of data governance policies and practices.

## FAIR in the Context of Indigenous Data

RDA as a truly global organization stands for data sharing without barriers. A significant contribution of RDA is to provide a platform for research communities, including for small, technical, or even sporadic ones. Researchers can participate in discussions at a global level, from their local research environment. Personal contributions on dedicated topics, via collaborative work results in agreed outputs and working methods.

The International Indigenous Data Sovereignty Interest Group, among many activities, created the CARE Principles and now seeks to identify policy and practice to implement the principles across data ecosystems. The CARE Principles, beyond supporting Indigenous Peoples’ rights and interests, is a new, game changing perspective, stimulating researchers across domains and regions as an effort to articulate community data rights.

The work on FAIR Principles and Open Science in recent years made it clear for both researchers and policymakers, that FAIR and Open are two interdependent issues, which are related but not synonymous^18^. FAIR is a technical removal (or lessening to a certain level) of the barriers, to make data interoperable, independent from the source, domain, or underlying technology. On the other hand, openness is a legal term connected to the rights of the data concerned. Some rights are well known and regulated (ownership), others are less regulated and more controversial. The role of personal data rights is clear and legislated in most countries, but due to the cultural legislative differences across regions and countries, there are no consistent rights. Both open and non-open datasets can be FAIR.

Community data rights are just at the beginning of a similar journey. The path provided by Indigenous Peoples in the data governance and policy field, will soon be followed by others who would like to give a voice to their collective rights. The OCAP® principles (ownership, control, access, and possession) were developed to recognise the collective rights of First Nations communities in Canada to information collected from their territories (https://fnigc.ca/OCAP). OCAP®, a registered trademark of the First Nations Information Governance Centre (FNIGC), enabled First Nations to assert control over data collection processes in their communities^19^, as well as recognise the authority to own, protect, and control how their information is used. The claiming of rights to own, control, access, and possess information about their peoples is fundamentally tied to First Nations self-determination and to the preservation and development of their culture. The OCAP® principles are supported by the FNIGC which has developed a First Nations Data Centre (FNDC) to provide access to unpublished and record-level data from FNIGC led survey work, including the First Nations Regional Health Survey and the First Nations Regional Early Childhood, Education and Employment Survey (https://fnigc.ca/fndc).

## Where to From Here․…

The articulation of the CARE Principles offers an opportunity to find synergies between the FAIR and CARE Principles with actions and responsibilities across the data lifecycle and ecosystem. Here we detail a preliminary set of recommendations for the data community to operationalise FAIR with CARE.

First, make Indigenous data FAIR. The FAIR criteria should apply to already existing and newly created Indigenous data in both Indigenous and non-Indigenous/hybrid datasets that mix Indigenous and non-Indigenous data. However, the use of Indigenous data in hybrid datasets requires a machine-readable provenance for Indigenous data and to signal the decision-making point that needs to be approached to allow or refuse consent to use the data. For Indigenous collections, Indigenous researchers and communities could try out the FAIR Data Maturity Model criteria, placing them at the forefront of the latest work on FAIR and assisting in identifying both actions to take to make existing data FAIR and to create policies and practices to make future data FAIR. In hybrid datasets there is a need to engage with Indigenous researchers and communities to co-produce policies and practices that can rectify the unFAIRness of existing data and ensure the FAIRness of newly created or deposited data alignes with the CARE Principles.

**Fig. 2:**
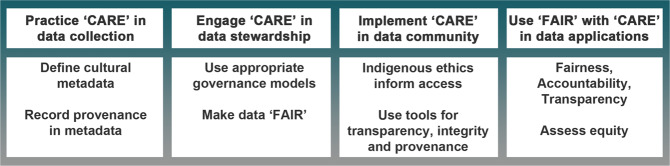
Implementation of the CARE Principles across the data lifecycle.

Second, proactively socialize data and research communities with the CARE Principles. These global to local communities should be aware of the need to apply the CARE Principles, and the actions to take before engaging with Indigenous data anywhere along the data lifecycle. While full adoption of the CARE Principles may be impeded while implementation criteria are created, there are clear actions to take and explore (see Fig. 2) such as ESIP’s efforts to apply CARE to repositories. For instance, practicing CARE in data collection requires defining cultural metadata and recording provenance. Engaging CARE in data stewardship necessitates using appropriate governance models and making data FAIR. Less clear and worthy of exploring are how to implement CARE in the data community and how to use FAIR with CARE in data applications. It would be regretful for Indigenous data to be misused. It is equally regrettable that Indigenous data are left aside due to lack of identifiers such as provenance or attribution metadata or unfamiliarity with interacting with Indigenous Peoples or their data. Exclusion of Indigenous data essentially erases Indigenous Peoples and interests from data related futures due to the perceived additional work because of uncertainty. Former would be unfair, while the latter would result in biased data solutions. The solution should be simple and deliverable even in an automated machine-readable environment.

Third, operationalise CARE for Indigenous Peoples’ data. There is a need to develop criteria and tools to implement the CARE Principles with FAIR. It took a long time and a lot of work and broad consultation to move from the FAIR Principles to FAIR Data Maturity Model criteria. Similar work could be done for CARE to achieve the consensus and agreement on the practical implementation. The working method and experience of the FAIR Data Maturity Model Working Group could support GIDA and the International Indigenous Data Sovereignty Interest Group to develop the CARE criteria with broad community input, which was used in both the drafting of the CARE Principles and the FAIR Data Maturity Model.

Fourth, utilize Indigenous design to benefit mainstream data communities. The CARE Principles are the first attempt to outline collective rights as part of openness. We caution the co-optation of the CARE Principles into other spaces just yet. As their full criteria for implementation have not yet been determined and used, we must leave space for the design and maturity of the CARE Principles to occur within the Indigenous environments from which they originate. While we recommend the time and space for future design, we fully recognize the benefits of using that Indigenous innovation as a best practice to develop similar principles/criteria for other communities with collective rights e.g., minoritized groups, geographically located communities, special professions, or consumers. This could be aligned to FAIR, if the “decision point” described in the first point can be identified and approached in an automated, machine-actionable way.

The discussion at the joint sessions underscored the need for Indigenous data to be both FAIR and CARE, from data creation to data reuse. FAIR and CARE are complementary perspectives which enable maximum value through the appropriate and ethical reuse of Indigenous data. However, assessing the FAIR-ness of a data set is typically a technical exercise which can be done independently by the researcher to prepare the final data set for reuse. On the other hand, the CARE Principles require engagement with people to address the cultural, ethical, legal, and social dimensions associated with the intended uses of the dataset. As Indigenous communities expect CARE-full data practices to be enacted at each step of the data lifecycle, we will need to reflect a broader temporal dimension to our application of the CARE Principles. At present there is no process to assess whether a research project meets the CARE Principles. Creating such an assessment represents the next stage towards an equitable cyberinfrastructure that supports the FAIR and CARE-full use of Indigenous data.
